# Analysis of addiction craving onset through natural language processing of the online forum Reddit

**DOI:** 10.1371/journal.pone.0301682

**Published:** 2024-05-20

**Authors:** Thea Kramer, Georg Groh, Nathalie Stüben, Michael Soyka

**Affiliations:** 1 Department of Informatics, Technical University of Munich, Munich, Germany; 2 Department of Psychiatry, Ludwig-Maximilians-University of Munich, Munich, Germany; NYU Grossman School of Medicine: New York University School of Medicine, UNITED STATES

## Abstract

**Aims:**

Alcohol cravings are considered a major factor in relapse among individuals with alcohol use disorder (AUD). This study aims to investigate the frequency and triggers of cravings in the daily lives of people with alcohol-related issues. Large amounts of data are analyzed with Artificial Intelligence (AI) methods to identify possible groupings and patterns.

**Methods:**

For the analysis, posts from the online forum “stopdrinking” on the Reddit platform were used as the dataset from April 2017 to April 2022. The posts were filtered for craving content and processed using the word2vec method to map them into a multi-dimensional vector space. Statistical analyses were conducted to calculate the nature and frequency of craving contexts and triggers (location, time, social environment, and emotions) using word similarity scores. Additionally, the themes of the craving-related posts were semantically grouped using a Latent Dirichlet Allocation (LDA) topic model. The accuracy of the results was evaluated using two manually created test datasets.

**Results:**

Approximately 16% of the forum posts discuss cravings. The number of craving-related posts decreases exponentially with the number of days since the author’s last alcoholic drink. The topic model confirms that the majority of posts involve individual factors and triggers of cravings. The context analysis aligns with previous craving trigger findings related to the social environment, locations and emotions. Strong semantic craving similarities were found for the emotions boredom, stress and the location airport. The results for each method were successfully validated on test datasets.

**Conclusions:**

This exploratory approach is the first to analyze alcohol cravings in the daily lives of over 24,000 individuals, providing a foundation for further AI-based craving analyses. The analysis confirms commonly known craving triggers and even discovers new important craving contexts.

## Introduction

In 2021, 3% of the German population were affected by alcohol use disorder (AUD). Craving is a key symptom for substance use disorders both in ICD-10 and DSM-5 [1] and multiple lines of evidence suggest craving for alcohol to be associated with risk for relapse [2, 3]. These cravings entail a strong desire to consume alcohol and are prevalent among the majority of individuals with AUD especially during the withdrawal phase and early abstinence. Cravings can be provoked by a variety of factors, including external factors such as specific locations, situations, and times of the day, as well as internal triggers like positive or negative emotions and mood states. Higher relapse rates have been associated with an increased craving frequency [4]. Despite the crucial role of coping with cravings in the treatment of AUD, there is still no conclusive concept or understanding of their causes and triggers [5]. The identification of risk and tempting situations and triggers may offer chances to develop better coping strategies to reduce the urge to drink (relapse prevention).

Over the past decade, multiple studies have linked cravings to individual and multidimensional contexts, and indicated their susceptibility to personal factors [6–13]. Some authors distinguish between reward craving (reinforcing effects of alcohol in positive situations, e.g. a party) and relief craving (avoidance of unpleasant effects, such as withdrawal symptoms). Predominantly negative moods such as stress, anxiousness, anger or a depressive mood can trigger cravings [6, 7, 14–24]. The affected person’s location and environment have also been found to impact general cravings, e.g. bar or restaurant related places [7, 13, 25]. In addition, nicotine use has been specifically linked to alcohol cravings [6, 7]. Cited previous studies are primarily based on surveys or experiments on artificially induced cravings through visual stimuli or virtual reality, with participant numbers generally not exceeding a few hundred individuals.

Limited research has been conducted on addiction within naturally occurring environments. Novel AI-based research strategies can help to elucidate the interrelationship between craving and relapse to alcohol. In the field of text mining, Chen et al. [26] and Cohn et al. [27] explored addiction to smoking in online communities. Chen et al. specifically focused on electronic cigarette use in various online forums and utilized Latent Dirichlet Allocation (LDA) topic models to demonstrate that smoking cessation varies depending on the e-cigarettes usage. Cohn et al. examined the topics, sentiment, and social network centrality of alcohol in an online smoking forum [27]. Their analysis of 6000 posts could be divided into three topics and mainly conveyed a nonnegative sentiment about alcohol.

Two studies related to alcohol addiction employed Ecological Momentary Assessment (EMA), a technique that collects data from participants’ daily lives, often through smartphone applications. Dulin et al. [28, 29] developed the “Location-Based Monitoring and Intervention for Alcohol Use Disorders” (LBMI-A) application as an intervention strategy for individuals with AUD. The application was utilized by 28 AUD patients over a six-week period, revealing significant associations between craving cues, craving strength, coping strategies, and post-craving drinking. Notably, easy access to alcohol and the time of day were identified as the most influential cues in succumbing to cravings. Kuerbis et al. [14] recently employed an EMA approach to capture relevant drinking contexts among heavy drinkers after collecting personalized drinking triggers for each of the 153 participants. Participants reported their craving level and context three times a day for 84 days. The primary findings indicated that previous drinking, negative affect, and familiar drinking settings exhibited strong associations with cravings. Generally, previously defined personal triggers were more strongly associated with intense and frequent cravings than general triggers. Additionally, the study revealed a direct relationship between greater AUD severity and increased odds of experiencing cravings.

To date, no study has investigated alcohol cravings on a large scale, particularly concerning their contexts and triggers. Therefore, this study aims to gather information about the triggers and reasons behind naturally occurring cravings from a substantial subject group, regardless of the severity of AUD.

## Materials and methods

Online communities offer a vast repository of first-hand experiences that provide an unfiltered authenticity due to the anonymity they offer. Leveraging text-mining and natural language processing (NLP) tools enhanced through AI enables the analysis of a large volume of posts. In this study, we aim to explore craving triggers by analyzing the mentions and contexts of cravings within the largest online community dedicated to individuals with AUD. The present investigation focuses on the popular online platform Reddit, which comprises a diverse range of Subreddits covering various topics. Specifically, our attention is directed towards the Subreddit “stopdrinking,” where more than 400,000 registered users (status 2022) can participate in discussions about AUD by sharing posts and comments while maintaining anonymity.

We utilized the Reddit API interface PushShift [30] to extract posts from the stopdrinking Subreddit. After manually scanning through several post samples, we identified a range of discussed topics, including first onset of AUD, daily life with AUD, relapses and cravings, and sharing of tips and motivational posts about sobriety. The number of posts on “stopdrinking” ranged from 150 to 200 daily, with an increasing number of posts and authors over time. We scraped posts from April 2017 until April 2022, excluding comments. To focus on posts that provide detailed descriptions, we removed deleted texts and short posts containing less than 60 characters. The resulting dataset consisted of 279,688 instances, which we further filtered for posts related to cravings.

### Craving filter

At the time of the study, no large, labeled dataset for cravings was published or available; thus, we could not rely on a learning algorithm for filtering relevant posts. To identify craving posts and their relevant contexts, we employed a regular expression (Regex) query that searches for character patterns in a text. The query consisted of synonyms for cravings that were both collected from craving papers and computed through the most similar words to ‘craving’ in our dataset. The synonyms were ‘craving’, ‘trigger’, ‘relapse’, ‘urge’, ‘desire’ and ‘temptation’, including their prefixes, suffixes, conversion and compound words. The word similarities were calculated via Gensim word similarity scores transforming the words into vectors called word embeddings with the word2vec technology (a common technique in AI for NLP).

The final Regex query retrieved approximately 16% of all posts as craving posts, or nearly every sixth post. The resulting craving dataset contained 44,920 posts by 24,435 distinct authors, with an average of 105 words per post. Notably, over 70% of the authors wrote only one post in the craving dataset, while almost 15% created two posts.

### Subjects

The approximately 25,000 post authors represent our study subjects who had all joined the Subreddit ‘stopdrinking’ as registered users on Reddit at the time of posting. ‘stopdrinking’ is described as a ‘place to motivate each other to control or stop drinking’ and the users are asked to exclusively create posts in a sober state [31]. The users are anonymous which allows no inference of gender, age or whether they have been diagnosed as having AUD. Hence, Reddit’s user base might not reflect the real world distribution in terms of diversity. By examining a sample of posts, we extrapolated that users from different geographical regions contribute to the discussions, with a notable representation from North America. Reddit data is publicly available and frequently used in research [26, 27, 32]. Since the data is freely available, anonymous, and we do not quote usernames or posts, this study is exempt from ethics approval.

### Topic modeling

**Fig 1:**
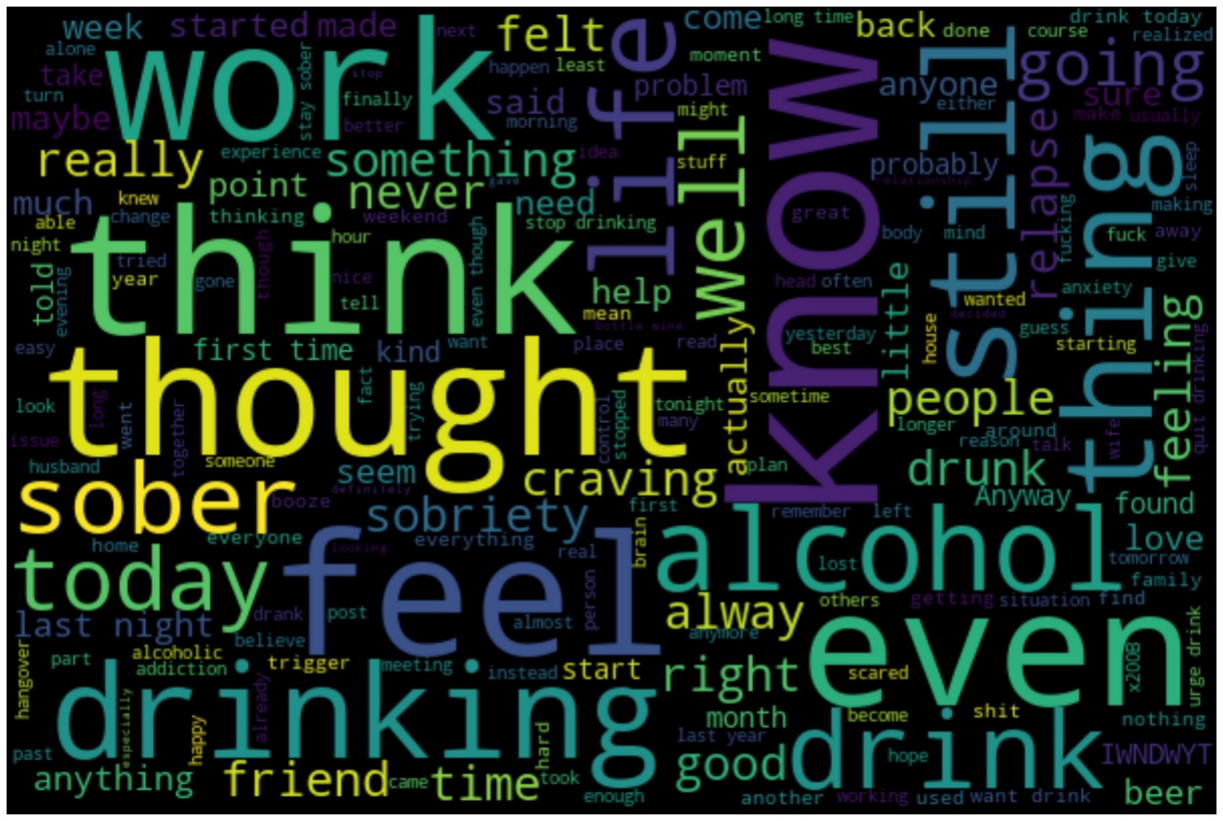
The most mentioned words for all craving posts in a word cloud. Words that appear more frequently are larger.

An overview of the most frequent words mentioned in the craving posts is displayed in the word cloud in Fig 1 which was generated with the python package ‘wordcloud’ [33].

Neglecting words that are generally used with a high frequency, we detected numerous words directly related to drinking (’alcohol’, ‘drinking’, ‘drunk’, ‘drink’, ‘sober’, ‘craving’, ‘beer’). Terms that could indicate craving contexts were ‘work’, ‘friend’, ‘happy’, ‘hour’, ‘family’, ‘meeting’, ‘scared’, ‘husband’, ‘alone’, ‘night or ‘weekend’. To obtain a more specific overview, we applied topic modeling to compute the most common themes, comparable to the approaches of Cohn et al. and Chen et al. [26, 27]. For clustering the topics, Latent Dirichlet Allocation (LDA) was used [34], a generative AI-model that is used to cluster text into themes by calculating lists of words that are representative for each theme [34, 35]. These word lists are then manually interpreted as a topic by finding an umbrella term. Before training the model, we preprocessed the craving data by lowercasing, tokenizing, and lemmatizing the text and removing stopwords and punctuation. We then used a bag of words (BOW) model to represent each post as a collection (bag) of words without order, which we fed into the LDA model. We selected 10 as the predefined number of topics based on common LDA metrics (the lowest validation perplexity and the highest coherence score).

### Craving contexts

We made the hypothesis that words and concepts that are frequently mentioned in craving posts relate to cravings and are more likely to represent (possibly subconscious) craving reasons or triggers than less frequently mentioned words based on the ‘distributional hypothesis’ in linguistics [36, Chapter 6]. In the craving dataset, we examined the occurrence of locations, social company, emotions, times of the day and week as well as nicotine consumption, all of which we derived as common factors for craving triggers from psychological research. Following the procedure for detecting craving posts, we used the Gensim similarity scores to identify the most similar terms for each category and manually curated the resulting lists to include only synonyms. The curation resulted in the following list queries:

- home: [‘home’, ‘house’, ‘bed’, ‘kitchen’, ‘living room’]
- university/school: [‘uni’, ‘college’, ‘school’, ‘campus’, ‘classroom’]
- work: [‘work’, ‘working’, ‘wfh’, ‘worked’, ‘job’, ‘office’, ‘coworkers’, ‘shift’]
- restaurant/bar: [‘restaurant’, ‘bar’, ‘cocktail’, ‘menu’, ‘server’, ‘waitress’]
- party: [‘party’, ‘festivity’, ‘gathering’,‘wedding’, ‘reunion’]
- workout: [‘workout’, ‘gym’, ‘exercise’, ‘routine’, ‘crossfit’, ‘cardio’, ‘jog’, ‘yoga’, ‘fitness’]
- supermarket: [‘supermarket’, ‘aisle’, ‘grocery’, ‘store’, ‘checkout’, ‘cashier’, ‘cart’, ‘walgreens’, ‘711’, ‘deli’, ‘walmart’]
- airport: [‘airport’, ‘layover’, ‘flight’, ‘plane’, ‘airline’, ‘delay’]
- anxious/worried: [‘anxious’, ‘sad’, ‘antsy’, ‘panicky’, ‘restless’, ‘tense’]
- sad: [‘sad’, ‘lonely’, ‘depressed’, ‘disappointed’, ‘hopeless’, ‘cry’]
- stressed: [‘stress’, ‘overwhelmed’, ‘overworked’, ‘unsettled’, ‘workload’]
- tired: [‘tired’, ‘exhausted’, ‘sick’, ‘frazzled’, ‘sluggish’, ‘drained’, ‘groggy’]
- frustrated/angry: [‘frustrated’, ‘irritated’, ‘snapping’, ‘pissy’, ‘mad’, ‘cranky’, ‘grumpy’, ‘ugh’]
- happy: [‘happy’, ‘excited’, ‘amazing’, ‘awesome’, ‘enjoying’, ‘grateful’]
- proud: [‘proud’, ‘accomplished’, ‘milestone’, ‘longest’]
- bored: [‘bored’, ‘boredom’, ‘boring’, ‘unmotivated’, ‘monotony’]
- alone: [‘alone’, ‘isolated’, ‘lonely’, ‘cooped’, ‘quarantining’]
- friend(s): [‘friend’, ‘buddy’, ‘pregaming’, ‘groomsman’, ‘friendsgiving’, ‘bros’]
- family: [‘family’, ‘parent’, ‘dad’, ‘brother’, ‘mother’, ‘wife’, ‘sister’, ‘mom’]
- partner: [‘partner’, ‘girlfriend’, ‘husband’, ‘boyfriend’, ‘wife’, ‘ex’, ‘bf’]
- colleague(s): [‘colleague’, ‘coworkers’, ‘manager’, ‘networking’, ‘boss’, ‘meetups’, ‘supervisor’]

When searching for these keywords, We grouped the posts by user and concatenated them to obtain a precise count of distinct users experiencing certain contexts or triggers. As there exists no dataset with craving-specific location labels at the time of publishing, we could not apply more advanced supervised machine learning techniques.

### Test set creation

We evaluated our calculations on two test sets (*set*_200_ and *set*_350_) which three human labelers manually labeled for 200 and 350 randomly sampled posts, respectively. Consistent labelling was ensured through an agreed set of definitions. We allowed multi-labels for the features social company, locations and emotions since for these features multiple features can occur in the same post. In more than 95% of the cases, the labelers were in consent, which is considered a high consensus.

## Results

### General metrics

**Fig 2:**
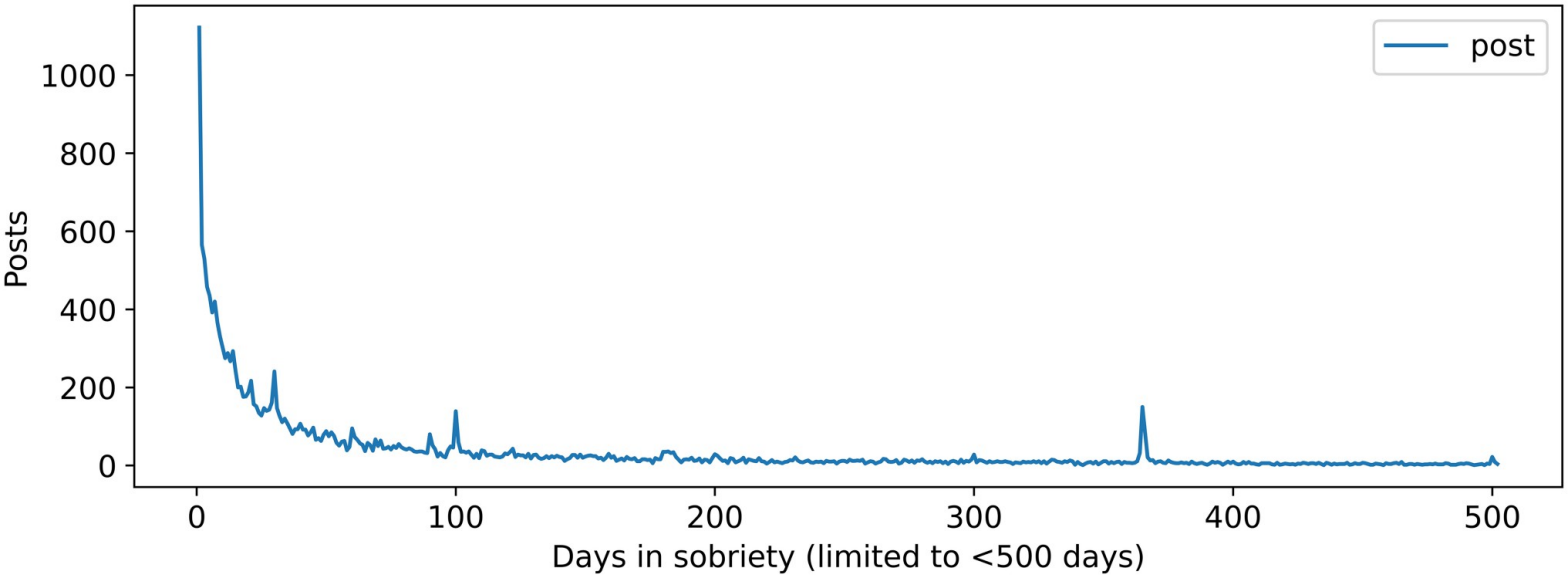
The number of posts created in the craving dataset related to the number of days in sobriety (retrieved from the author flair text). For readability, the graph is only displayed until the first 500 days.

In the context of the studied online forum, post authors can optionally activate a badge that counts how many days ago their last alcohol consumption took place since the post publishing (author flair text). The badges from the craving set ranged from one day to 14,662 days (approximately 40 years). Noticeably many posts were created on milestone days for the author, such as after one month, after 100 days or after one year since the last alcoholic drink (visible in Fig 2).

When looking at the distribution of craving posts depending on the author flair text in Fig 2, we noticed that cravings were mentioned at all stages of AUD, however with an exponential decrease with the number of days since quitting alcohol.

**Fig 3:**
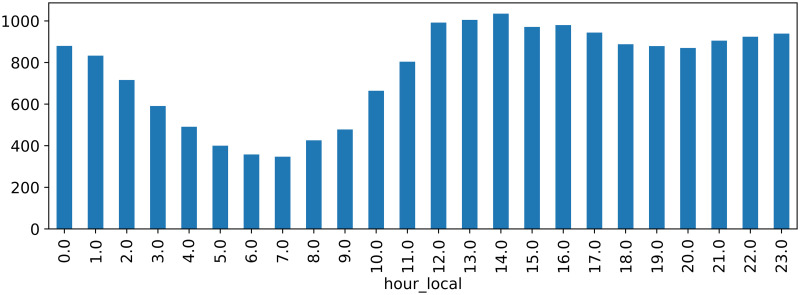
Number of craving posts per hour.

Concerning time-related peaks, we evaluated the time of the day, week and year of craving posts that were part of the API data. The craving posts were roughly evenly distributed over the months and days, and no peaks occured during weekends or specific holidays, even though these were mentioned triggers in a few posts. Within the hours of the day, a significant increase of activity could be noticed in the afternoon until late evening (roughly 1pm until 11pm) in comparison to the night and morning (calculated in the users’ local time to avoid distortion), see Fig 3.

Considering the word distributions of time related cues, weekend days or the term ‘weekend’ was mentioned almost twice as much as the other weekdays together, specifically, more than 26% of authors included it in their craving posts. Also, ‘Friday’ and ‘Saturday’ were significantly more similar to the term ‘craving’ than the other weekdays (37% and 40% compared to 23%-31%) which means they were semantically more closely related to cravings. Similarly, evening and nighttimes appeared with notable frequency; nearly 44% of authors mentioned it in context with cravings, whereas ‘morning’ and afternoon were mentioned by roughly 20% and 6.6%, respectively. However, the word similarity scores of ‘night’ and ‘afternoon’ to the term ‘craving’ were both notably higher (more than 41%) than the similarities of ‘craving’ to ‘midday’, ‘morning’ or ‘lunch’ (less than 37%). This relates to our findings from Fig 3. Nicotine, smoking and tobacco-related terms appeared in 12.02% of the craving posts grouped by author. Reading sample posts, nicotine was mentioned both as a trigger for alcohol cravings and as a countermeasure. 14.1% of the authors talked about depression in the craving posts and 23.68% about anxiety.

When computing the most similar terms in our craving set, we find that ‘crave’ was listed as one of the most similar terms to ‘bored’ with 45% similarity. Furthermore, ‘stressed’ had ‘drink’ listed within the highest similarities (40%). Another finding is that airport has a high similarity to the word ‘trigger’ with 46%.

### Topic modeling

Table 1 displays the topics from the LDA modelling together with their keywords (words with the highest probability), their topic names derived through human judgement, the coherence scores and the percentage of distinct words that appear in the dataset and belong to the respective topic. The coherence score measures the similarity score between the top scoring words per topic using normalized pointwise mutual information (NPMI). In the third column of the table, we selected the twelve keywords with the highest probability for each topic, omitting words that occur equally frequently in other categories.

**Table 1:** The 10 topics clustered by the LDA.

#	Our summary	Words with highest probability	Coh.	%w.
1	Life story	‘time’, ‘know’, ‘want’, ‘year’, ‘make’, ‘think’, ‘sober’, ‘thing’, ‘life’, ‘try’, ‘help’, ‘way’	-0.91	38.7
2	Emotions and moods	‘day’, ‘feel’, ‘week’, ‘last’, ‘craving’, ‘feeling’, ‘anxiety’, ‘sleep’, ‘strong’, ‘tired’, ‘energy’, ‘awful’	-1.03	22.6
3	Daily life at home	‘home’, ‘tell’, ‘leave’, ‘fuck’, ‘tonight’, ‘call’, ‘drive’, ‘wife’, ‘watch’, ‘kid’, ‘house’, ‘room’	-1.63	12.8
4	(Social) craving triggers	‘drink(ing)’, ‘night’, ‘beer’, ‘alcohol’, ‘friend’, ‘stop’, ‘drunk’, ‘weekend’, ‘wine’, ‘fun’, ‘enjoy’, ‘party’	-1.84	10.3
5	Real-life Support	‘meeting’, ‘experience’, ‘learn’, ‘share’, ‘step’, ‘other’, ‘group’, ‘read’, ‘book’, ‘recovery’, ‘program’, ‘side’	-2.14	5.5
6	Duties and burdens	‘job’, ‘move’, ‘money’, ‘pain’, ‘parent’, ‘die’, ‘due’, ‘kill’, ‘lose’, ‘school’, ‘pay’, ‘young’	-2.53	3.3
7	Craving distractions	‘eat’, ‘amp’, ‘food’, ‘weight’, ‘coffee’, ‘water’, ‘smoke’, ‘here’, ‘exercise’, ‘busy’, ‘sugar’, ‘cigarette’	-2.96	2.3
8	Addiction as a disease	‘addiction’, ‘drug’, ‘alcoholism’, ‘addict’, ‘abuse’, ‘smoking’, ‘blow’, ‘high’, ‘substance’, ‘history’, ‘addicted’, ‘worker’	-3.40	1.7
9	Online support	‘re’, ‘perfect’, ‘sex’, ‘stopdrinke’, ‘mindset’, ‘badge_reset’, ‘card’, ‘participate’, ‘app’, ‘stopdrinking_comment’, ‘link’, ‘tackle’	-3.36	1.4
10	Medical treatment	‘doctor’, ‘com’, ‘medication’, ‘hospital’, ‘liver’, ‘symptom’, ‘treatment’, ‘mouth’, ‘key’, ‘appointment’, ‘pill’, ‘study’	-3.05	1.4

For each topic, the number, our chosen umbrella term (summary), the words that have highest probability to belong to that topic, the coherence score and the percentage of words that belong to that topic are shown.

For topics 2, 4, 5, 6, 7, 8 and 10, nearly all words aligned with our found umbrella term. The other topics were broader, yet they matched their summary terms as well.

Finding umbrella terms emerged naturally and intuitively, which is a successful outcome for topic modelling.

### Craving filter and contexts

To evaluate the contexts of the craving set, we first assessed the effectivity of our craving filter. We randomly drew 200 posts from the base dataset (*set*_200_), of which 29 were identified as craving posts by our query and 41 were labeled as craving posts by at least two of the three human labellers. Given that false positives are more detrimental than false negatives in our case, we prioritized precision over recall, achieving an accuracy of 89% and a precision of 82.76%. Precision measures the ratio of true positives divided by the sum of true positives and false positives, whereas recall stands for the ratio of true positives divided by the sum of true positives and false negatives.

Our feature analysis and evaluation on *set*_350_ are presented in Table 2.

**Table 2:** Results of the craving set and test set.

Feature	% authors in craving set (predicted)	% in *set*_350_ (predicted)	Accuracy	Precision	Recall
craving	100.00	100.00	86.86	86.86	-
with partner	36.41	19.94	95.71	94.29	85.71
with family	35.83	22.51	92.29	86.08	80.95
with friend(s)	33.62	21.43	94.86	85.33	90.14
alone	18.06	12.25	92.57	69.77	69.77
with colleagues	3.71	2.28	95.71	62.50	29.41
at work	49.61	39.32	80.86	55.07	93.83
at home	38.99	25.93	91.43	84.62	82.80
workout	18.90	11.11	92.57	69.23	65.85
at a bar/restaur.	16.74	9.69	96.29	73.53	86.21
in a store	16.07	10.54	93.14	48.65	78.26
at a party	13.00	8.55	95.71	83.33	71.43
at school	9.81	6.84	97.14	58.33	100.00
in an airport	2.14	1.14	99.14	50.00	66.67
anxious	45.01	31.91	86.29	83.04	76.23
happy	35.54	26.21	83.43	63.04	70.73
sad	30.44	19.09	87.43	79.10	63.86
frustrated	26.19	16.24	77.43	57.89	37.50
proud	18.58	12.25	86.29	76.67	35.94
tired	18.11	11.97	92.29	57.14	72.73
stressed	16.76	12.82	94.00	71.11	80.00
bored	11.69	6.55	97.14	78.95	71.43

The craving set consist of posts grouped by author with almost 25,000 distinct authors. The *set*_350_ consists of 350 posts.

Generally, features occurred more frequently in the test set (column three) than in the craving set (column two), because the test set posts were not grouped and concatenated by author. However, this dataset difference has no impact on evaluating the accuracy of our queries.

The precision is similar to the recall in most cases, which means that the occurrence percentages in column two are likely close to the actual percentages. The test set generally shows good results for well-represented features, whereas features such as school and airport locations or the company of colleagues were prone to worse results due to their underrepresentation.

Classifying emotions is more complex than extracting locations or social company since one state of emotion can be composed of multiple emotions and sometimes merely hints of an emotion may be present. Hence, the evaluation metrics fluctuated more than for the other categories.

## Discussion

The primary objective of this study was to investigate the onset and frequency of alcohol cravings in real-world settings, its social, environmental and individual context and compare them to findings from laboratory studies. We contribute by analyzing compiled Reddit data from nearly 25,000 individuals who have experienced issues with alcohol. Our study surpasses previous research due to the large scale of the dataset and examining naturally occurring cravings rather than lab-induced ones, enabled by AI-powered NLP. The abundance of data and focus on real-world cravings enhance the robustness and applicability of our findings. As stated in the introduction, field data analyses of alcohol cravings had been previously conducted, however without the use of AI and with lower participant numbers [14, 28].

The main findings indicate that significantly more people communicate about cravings during the initial days of sobriety compared to later stages (as shown in Fig 2). Nonetheless, posts related to cravings were observed at all stages of sobriety.

The terms ‘stressed’, ‘bored’ and ‘airport’ were identified as highly associated with ‘drinking’, ‘craving’ and ‘trigger’, respectively. Similarly, cravings were found to be more closely linked to the evening, night and weekend compared to other times of the day and weekdays. Whereas the significance of daytime and weekdays as triggers was already known [7, 8], the identification of airports as a trigger is a novel finding. Furthermore, the identification of stress and boredom as the most prominent terms associated with drinking situations is also a novel discovery, as previous research mentioned these factors but not with such predominance [8, 21, 23]. Other craving contexts related to locations, social environments, and emotions, which were previously identified as craving triggers in research, were also present in the dataset. The study calculates the percentage of authors who used these terms, providing an understanding of the magnitude and impact of each trigger. This aspect is a novelty in comparison to previous studies, as it sets the frequency of triggers in relation to each other based on a high sample number. The analysis of the ten generated topic models sheds light on the various topics relevant to individuals with alcohol problems in the context of cravings. These models confirm that craving triggers, coping mechanisms, and daily situations involving cravings constitute the majority of topics within our craving dataset. Similar topic modeling approaches in the field of addiction had previously only been conducted for nicotine addiction [26, 27].

### Limitations

There are a few limitations to be discussed. First and predominantly, this is no clinical study or dataset—biases could be introduced through missing information on the sociodemographics or diversity of the participants, as explained in the Subjects section. Moreover, not all types of patients with AUD actively participate in Reddit, and no information about the severity of AUD is given for the posts. Furthermore, the truthfulness of each post cannot be verified, leading to a lack of confidence regarding the accuracy of statements. It is important to stress that the analyzed contexts cannot be automatically interpreted as craving triggers, but rather hint towards a possible correlation. Because of the above-mentioned limitations, results cannot be generally applied to all AUD patients. Still, Reddit serves as a valuable data source due to its non-clinical environment, allowing for a wide range of participants, including those who do not seek therapy or similar interventions. Due to the anonymity, the posts are likely phrased in a more intimate and authentic manner than in study settings.

For the context analysis, the percentages only show an approximation of the actual percentages, as shown in the test set validation. Especially for underrepresented contexts and for emotions, the precision and recall vary. However, since we are not interested in the results of single instances but rather the accumulated percentages, the predicted percentage values for contexts with a precision similar to recall are likely to represent the actual percentages. For a more advanced assessment of scarcely occurring contexts, more test set labels need to be created.

## Conclusion

In conclusion, the study shows that cravings are relevant particularly in the first days of sobriety but continue to appear in posts even after months or years. Most known craving triggers related to social surrounding, location or emotions were present in the dataset and specifically boredom, stress and airport were semantically strongly linked to craving related terms. The findings of the topic model provide empirical evidence that a substantial portion of the craving post dataset revolves around themes about craving triggers or situations.

For future research, it would be advantageous to identify patterns within craving categories instead of relying on a binary craving model (a post is about a craving or not). This approach would enable to study the intensity and kind of cravings. Additionally, refining the craving and context filter could enhance the recall and precision of the test set. Another interesting avenue for improvement would be the development of an AI-based approach that provides further insights into the demographics of the authors.
